# Supplementary material for: Short-Term l-arginine Treatment Mitigates Early Damage of Dermal Collagen Induced by Diabetes
Source: Bioengineering (Basel). 2024 Apr 21;11(4):407. doi: 10.3390/bioengineering11040407 (PMC11048012; doi:10.3390/bioengineering11040407)
Supplement: Supplementary file 1 [file bioengineering-11-00407-s001.zip › bioengineering-2938298-supplementary.pdf]

Supplementary Materials

Table S1

Table S1. Collagen I Raman band assignments for the spectral region 700-1800 cm^{-1}

Position	Assignation	Reference
**823 cm^{-1}**	C-C stretching of collagen backbone	[49]
**831 cm^{-1}**	Out of plane C-H collective motion in tyrosine	[37], [41]
**858 cm^{-1}**	Proline, hydroxyproline and tyrosine<br>C-C stretching of proline and hydroxyproline<br>Side chain vibrations of proline and hydroxyproline<br>C-C vibration of the collagen backbone	[45], [37]<br>[49], [36]<br>[35], [46]
**917 cm^{-1}**	C-C stretching of proline	[35]<br>[42], [36]
**934/937 cm^{-1}**	C-C stretching of collagen backbone	[44], [36]<br>[42], [45]<br>[46], [35]
**951 cm^{-1}**	Tyrosine out of plane vibration	[48]
**1008 cm^{-1}**	Phenylalanine	[49], [36]
**1035 cm^{-1}**	C-N stretching in proline<br>C–H in-plane bending mode of phenylalanine	[49]<br>[37]
**1067 cm^{-1}**	C-N stretching in proline	[49]
**1100cm^{-1}**	Deformation N-C-H in proline	[49]<br>[37], [36]
**1249 cm^{-1}**	Amide III<br>(C-N stretching of amide bond)	[49], [35]<br>[46], [36]
**1276 cm^{-1}**	Amide III	[36, [38]<br>[35], [47]
**1314 cm^{-1}**	CH_3CH_2 twisting-collagen	[38]
**1320 cm^{-1}**	CH_3CH_2 deformation of collagen<br>(CH_3CH_2 twisting in collagen)	[36], [47]<br>[35]
**1336 cm^{-1}**	CH_3CH_2 deformation of collagen<br>(CH_3CH_2 wagging in collagen; CH2 scissoring)	[37]<br>[35]
**1445 cm^{-1}**	CH_3CH_2 deformation of collagen<br>CH_2 bending and scissoring modes of collagen and phospholipids	[38]
**1465 cm^{-1}**	CH_2/CH_3 deformation of collagen and lipids<br>CH_2 wagging	[38]
**1544 cm^{-1}**	Amide II	[38]<br>[39]
**1557cm^{-1}**	Amide II<br>(Stretching C-N and deformation N-H)	[38]
**1594 cm^{-1}**	Phenylalanine	[38]<br>[40]
**1644 cm^{-1}**	Amide I	[38]
**1665 cm^{-1}**	Amide I	[38]<br>[36], [42]
**1680 cm^{-1}**	Amide I	[38]
**1700 cm^{-1}**	Amide I	[38]<br>[35]

Figure S1 and Figure S2

Non-negative PCA analysis was performed on two intervals: (700-1400) cm^{-1} and (1400-1800) cm^{-1} separately because the prominent modes in the latter interval are several times more intensive than the majority of modes in the first interval. First three loading vectors and corresponding PCA score pair plots for both intervals are shown in the Figure S1 and Figure S2.

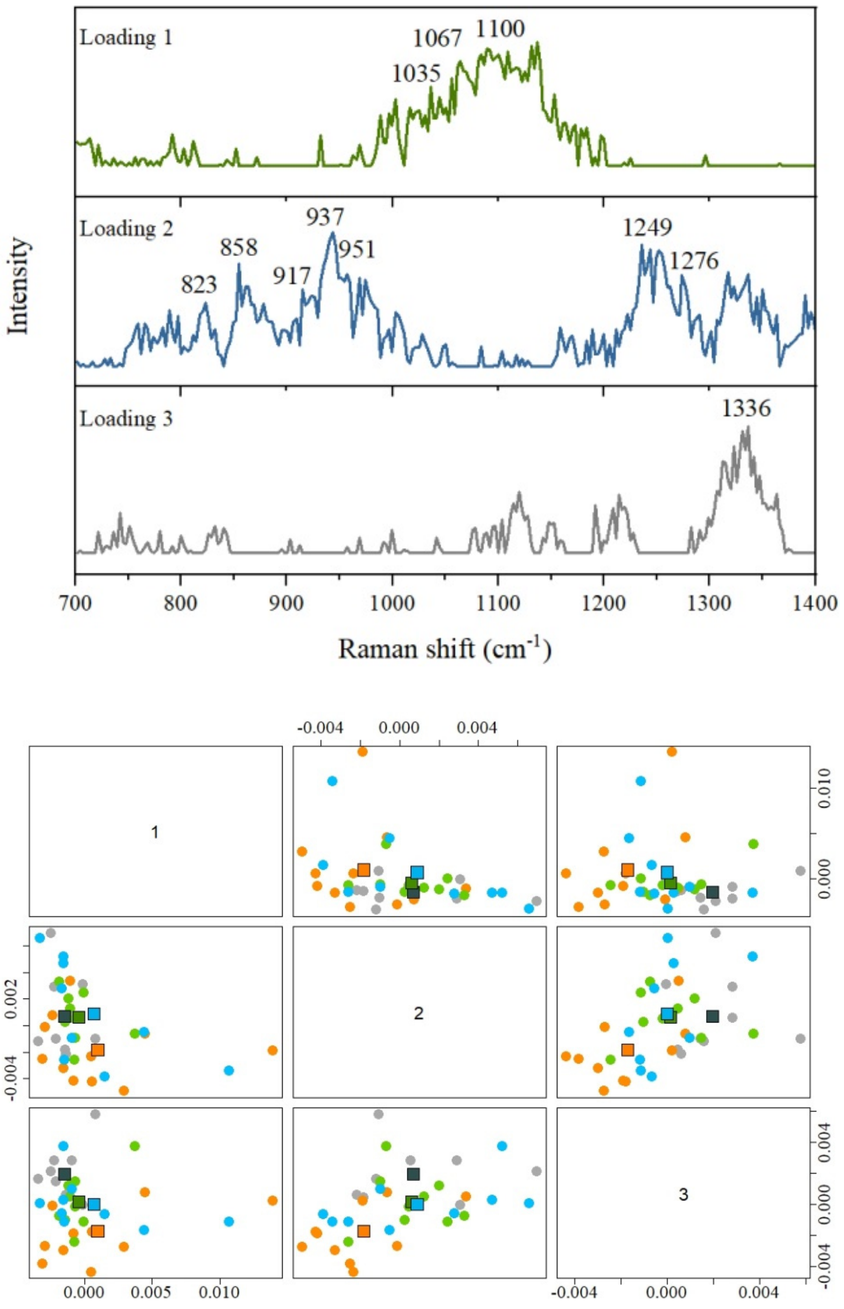

Figure S1. In the upper panel are presented PCA loading vectors 1-3 and in the lower panel score pair plots for the interval (700-1400 cm)$^{-1}$. Circles represent score values for each spectra from the experimental groups: control (non-diabetic) - *dark grey*, L-arginine (L-arginine-treated non-diabetic) - *sky blue*, diabetic - *orange*, and diabetic L-arginine-treated rats - *green*. Mean values of the scores for each group are represented by squares and colored as the group they correspond to.

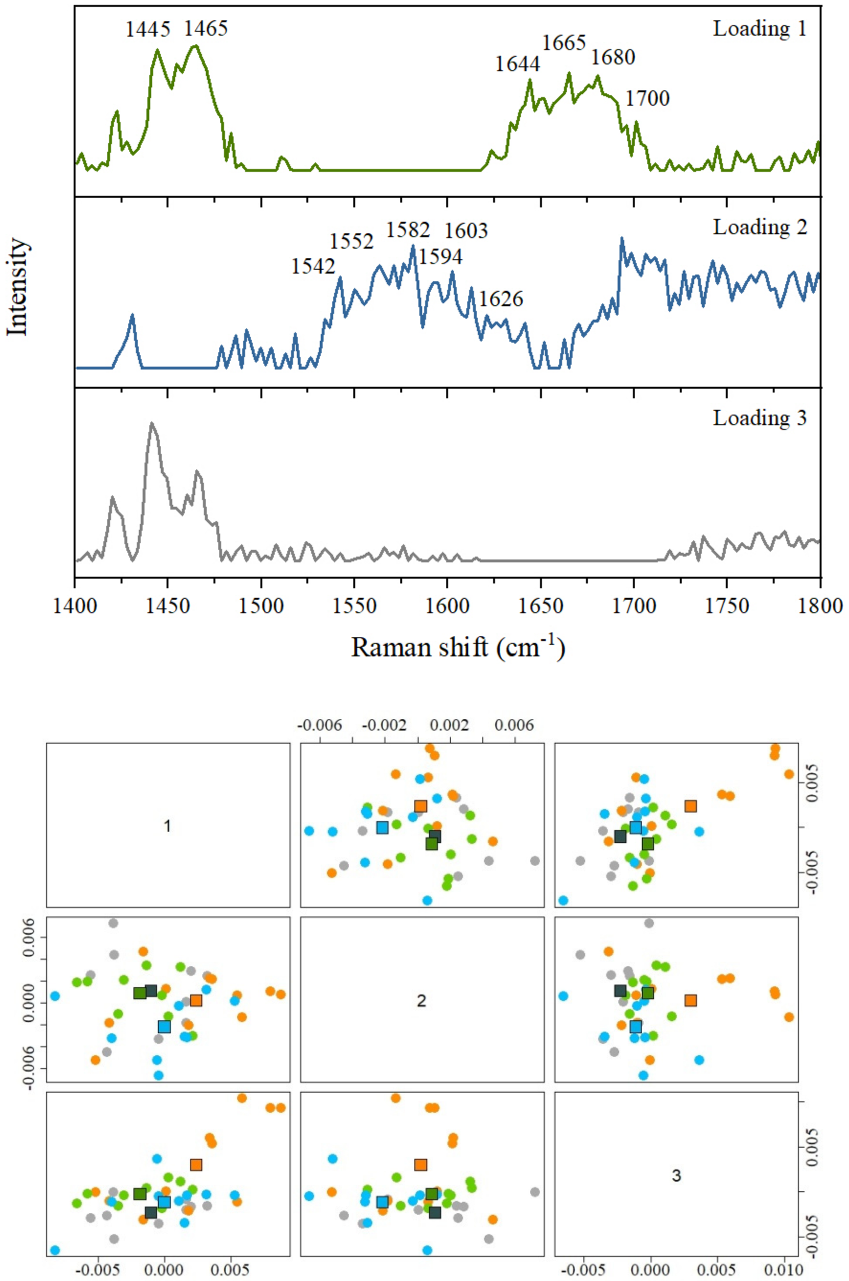

Figure S2. In the upper panel are presented PCA loading vectors 1-3 and in the lower panel score pair plots for the interval (1400-1800 cm)$^{-1}$. Circles represent score values for each spectra from the groups of samples: control (non-diabetic) - *dark grey*, L-arginine (L-arginine-treated non-diabetic) - *sky blue*, diabetic - *orange*, and diabetic L-arginine-treated rats - *green*. Mean values of the scores for each group are represented by squares and colored as the group they correspond to.

First loading vector in Figure S1 shows a broad peak with several superimposed peaks in the interval (1000-1200) cm^{-1}. In the score plot for the first component the means are ordered as following: control < diabetes+L-arginine < L-arginine < diabetes, which means that the intensity of the peaks shown in loading component one increases in the same order.

In a similar way the intensities of the vibrational modes positioned at 823 cm^{-1}, 858 cm^{-1}, 917 cm^{-1}, 937 cm^{-1}, 951 cm^{-1}, 1249 cm^{-1} and 1276 cm^{-1}, observed in the loading vector 2, increase in the order: diabetes < diabetes+L-arginine ~control < L-arginine.

The intensity of the 1336 cm^{-1} mode, observed in the loading vector 3 in Figure S1, increases in the order: diabetes < diabetes+L-arginine ~ L-arginine < control.

Features observed in the loading vector 1 in Figure S2 are positioned at 1445 cm^{-1}, 1465 cm^{-1} (CH_2 bending modes of proteins) and in the region 1620-1700 cm^{-1} (amide I modes) have intensities which increase in the order: diabetes+L-arginine ~control < L-arginine < diabetes.

By observing loading vector 2 and scores for component 2 (graphs in the middle column of lower panel) in Figure S2, it can be concluded that the modes positioned between 1580 cm^{-1} and 1630 cm^{-1} increase in the intensity in the order: L-arginine < diabetes < diabetes+L-arginine ~ control.
